# Editorial: Recent advancements in microbe-pesticide interaction: a smart-soil bioremediation approach

**DOI:** 10.3389/fmicb.2023.1206179

**Published:** 2023-05-17

**Authors:** Pankaj Bhatt, Shaohua Chen, Cormac Murphy

**Affiliations:** ^1^Agricultural & Biological Engineering, Purdue University, West Lafayette, IN, United States; ^2^State Key Laboratory for Conservation and Utilization of Subtropical Agro-Bioresources, Integrative Microbiology Research Centre, South China Agricultural University, Guangzhou, China; ^3^School of Biomolecular and Biomedical Science, University College Dublin, Dublin, Ireland

**Keywords:** microbiome, pesticides, interaction, environment, sustainability, toxicity

## Introduction

In recent years, there has been a growing interest in the development of eco-friendly solutions to control pests and promote soil health. One promising approach is the use of microbe-pesticide interactions to achieve smart-soil bioremediation (Bhatt et al., 2020). This approach involves the use of microorganisms that can degrade pesticides, detoxify the soil, and promote plant growth. Recent advancements in this field have shown great promise for sustainable agriculture (Wu et al., 2023). One of the most significant recent advancements in microbe-pesticide interaction is the discovery of new strains of microorganisms that can degrade a wide range of pesticides. These microorganisms can break down pesticides into harmless compounds, which helps to reduce their toxicity and prevent their accumulation in the soil. This not only benefits the health of the soil but also reduces the risk of pesticide contamination in food and water sources (Alexandrino et al., 2022).

Another key advancement in this field is the development of biopesticides, which are pesticides that are derived from natural sources, such as microorganisms, plants, or animals. Unlike conventional pesticides, biopesticides are non-toxic and do not harm the environment or non-target organisms. By using biopesticides in combination with microbe-pesticide interactions, it is possible to achieve effective pest control while promoting soil health. Moreover, recent studies have shown that microbe-pesticide interactions can promote plant growth and increase crop yield. Microorganisms can produce growth-promoting compounds that stimulate plant growth and enhance nutrient uptake. By promoting plant growth, microbe-pesticide interactions can help to increase the efficiency of crop production and reduce the need for chemical fertilizers (Glare and O'Callaghan, 2019).

Therefore, the recent advancements in microbe-pesticide interactions have opened new possibilities for sustainable agriculture. By using smart-soil bioremediation approaches that combine the use of microorganisms and biopesticides, it is possible to achieve effective pest control while promoting soil health and enhancing crop yield. These eco-friendly solutions can help to address some of the major challenges facing modern agriculture, such as pesticide resistance, soil degradation, and food security. As such, we must continue to invest in research and development in this field to ensure a sustainable and healthy future for our planet.

In this Research Topic, we have accepted 16 papers authored by 118 authors. The themes of the papers covered microbe-pesticide interactions, bioremediation and the response of gut microbiota to pesticides. The major highlights of the Research Topics are described below.

## Biodegradation of pesticides in environmental microorganisms

Qian et al. describes an investigation on the potential of using a combination of arbuscular mycorrhizal fungus (AMF) and *Hansschlegelia zhihuaiae* S113 to remediate soils contaminated with the herbicide bensulfuron-methyl. The researchers found that the combined use of AMF and *H. zhihuaiae* S113 was more effective in reducing bensulfuron-methyl residues in soil compared to the use of either organism alone. The study also showed that the combination treatment improved soil nutrient availability and microbial activity, which may have contributed to the enhanced biodegradation of bensulfuron-methyl. In another study, Kumar et al. suggested that the use of environment-restoring microbes isolated from the rhizosphere of horticultural crops under subtropics may provide a promising approach for the bioremediation of chlorpyrifos-contaminated soils. Sharma et al. identified the rhizospheric bacteria for the removal of the carbendazim in soil. They showed the inhibitory effect of carbendazim in isolated bacterial strain. They showed carbendazim (3,000 μg/mL) decreases the concentration of indole acetic acid, ACC deaminase and siderophore production in bacterial cells.

Li et al. suggest that the L1 microbial consortium has the potential to be used for the bioremediation of chlorimuron-ethyl-contaminated soils. However, further research is needed to optimize the conditions for the degradation process and to assess the long-term effectiveness and safety of this approach. The findings of this study may also have implications for the development of microbial consortia for the bioremediation of other herbicides and pollutants. The identification and characterization of the novel dmdA gene and its product may also have implications for the development of new herbicide-resistant crops and the biotechnological production of valuable compounds from dicamba. Zong et al. reported on the novel pyrethroid degrading enzyme SGNH esterase Est882, which can degrade diverse pyrethroids significantly.

Singh and Saxena discussed the recent findings on the pesticide-microbe interaction and showed the relationship among environmental factors at various trophic levels. He et al. discovered the cypermethrin-degrading binary consortium of the genus *Rhodococcus* and *Comamonas*. The developed consortium was effective for the elimination of pyrethroids from the environment. Wahla et al. reported that biochar-immobilized bacteria enhanced metribuzin degradation in the environment. Similarly, Pan et al. observed that immobilized bacteria can accelerate atrazine degradation in soil. Zhao et al. suggested development of the solid agents can facilitate the soil microbial degradation of pesticides.

## Toxicity of pesticides in living systems

Baazeem et al. reported *Bacillus subtilis* and other bacterial strains identified in their study can be used as an alternative to chemical pesticides for the control of *Aphis punicae* and *Aphis illinoisensis*. However, further research is needed to evaluate the efficacy of these bacterial strains under field conditions and to assess their long-term impact on the environment. Siddique et al. highlight the importance of insect gut microbiota in the degradation of pesticides. The gut microbiota of insects can play a critical role in detoxifying pesticides and reducing the accumulation of pesticide residues in the environment. The gut microbiota can also modify the physicochemical properties of pesticides, making them more susceptible to degradation. The composition of gut microbiota in insects can be influenced by several factors, including diet, host genetics, and environmental conditions. Therefore, the gut microbiota of insects can adapt to the presence of pesticides, leading to the development of pesticide-resistant gut microbiota. Astaykina et al. reported the impact of three pesticides on the gut microbiota of a earthworm *Lumbricus terrestris*. Pathak et al. discussed the negative impacts of pesticides on the environment, and living systems. They suggested the adoption of more sustainable and eco-friendly management strategies using microbial technology.

Jaffer et al. described the the gut microbiota in insect physiology and the biodegradation of pesticides, and suggests that this knowledge could be leveraged for the development of more sustainable and eco-friendly approaches to pest management.

## Future research

The articles published on this Research Topic advanced our understanding of pesticide-microbe interactions in soil and other environments. The Research Topic focuses on the degradation of pesticides using various bacterial and fungal strains. In addition, the Research Topic gives insights into the impact of pesticides on the gut microbiota. Further work is needed to meet the major challenges in the field of microbe-pesticide interaction. Advances in 'omics technologies will promote discoveries of novel methods and future applications capable of cleaning large-scale polluted sites in an eco-friendly manner.

## Author contributions

The editorial draft was written by PB, SC, and CM. All authors contributed to the revision and improvements and approved the final version for submission. All the editors collaborated well for the successful completion of the Research Topic.
